# Ego depletion and its role regarding the attitudes and behavior toward sustainable food consumption

**DOI:** 10.3389/fnut.2025.1469301

**Published:** 2025-05-08

**Authors:** Fabian Daiss, Markus Siebertz, Petra Jansen

**Affiliations:** Faculty of Human Sciences, Institute for Sports Science, University of Regensburg, Regensburg, Germany

**Keywords:** ego depletion, self-control, explicit attitudes, implicit attitudes, vegetarian nutrition, sustainable behavior, dual-process models

## Abstract

**Objectives:**

The study’s main goal was to investigate the effect of ego depletion on explicit and implicit attitudes and behavior toward sustainable food consumption in the context of dual-process models describing sustainable behavior.

**Methods:**

171 student participants completed an explicit rating and an affective priming task, respectively, at pre- and post-intervention, namely a six-minute transcription task to induce ego depletion. They then conducted a decision-making task (sustainable vs. less-sustainable chocolate bar) to test sustainable behavior during ego depletion.

**Results:**

Contrary to our hypotheses, explicit attitudes toward sustainable nutrition remained stable across conditions, showing no significant decline in the depletion group. Unexpectedly, implicit attitudes toward sustainable vegetarian nutrition became more negative over time, irrespective of the experimental condition. In the decision-making task, participants’ behavior was primarily predicted by their explicit attitudes post-intervention, rather than their implicit attitudes or ego depletion state.

**Conclusion:**

These findings challenge the assumption that ego depletion weakens explicit attitudes toward sustainable behavior, particularly vegetarian nutrition. Instead, explicit attitudes appear to be stable and the predominant predictor of sustainable food choices.

## 1 Introduction

Individual human behavior and consumption significantly contribute to environmental challenges and socio-environmental crises (1). Sustainable consumption refers to individual acts of fulfilling personal needs across different areas of life without compromising the ecological and socio-economic conditions of people currently living or in the future to satisfy their needs (2). A key area of sustainable consumption is food choice. In particular, reducing meat consumption is one of the most effective individual actions to lower carbon emissions (3), reduce energy and water consumption, and prevent biodiversity loss (4). Beyond ecological concerns, plant-based nutrition offers notable health benefits, such as lower morbidity risks and a reduced prevalence of chronic diseases (5). Despite these advantages, sustainable food choices often require individuals to override immediate preferences in favor of long-term environmental and health benefits (6).

This study investigates the psychological mechanisms underlying sustainable food choices by focusing on the role of self-control. Self-control can be described as the effortful attempt to delay instant gratification by changing thoughts, feelings, and behaviors to interrupt undesired behavioral tendencies and to bring them into line with standards such as ideals, values, morals, social expectations and to reach long-term goals (7). It plays a crucial role in promoting sustainable consumption, as it is significantly associated with pro-environmental behavior (8, 9), reduces impulsive overeating, and supports healthy eating behavior (10). According to the strength model of self-control (7), it is a resource, that can be depleted by mentally exhausting tasks requiring self-control. The resulting psychological state is called ego depletion. This effect seems to be universal and not domain-specific and has been widely studied in the context of eating behavior, showing that ego depletion increases the likelihood of consuming high-calorie, unhealthy foods and reduces adherence to dietary restrictions (11, 12). Recent evidence has reframed ego depletion as a process of energy conservation rather than full exhaustion and supports its reliability in applied settings, such as health and sports behavior (13).

The physiological basis of ego depletion has been linked to neural mechanisms. Research suggests that self-control relies on the prefrontal cortex, which regulates goal-directed behavior and impulse control (14).

Following Bamberg and Möser (15), sustainable behavior is seen as a mixture of concern for other people and self-interest with several individual factors like values, awareness of consequences, personal and social norms, attitudes, intentions, and habits described in the Comprehensive Action Determination Model (16). Most of the literature describes behavior because of an individual’s rational decision based on their values, attitudes, or intentions without considering that these can also be unconsciously influenced by situational variables (17, 18).

Dual-process models, like the Affective-Reflective-Theory (ART; (19)), use two different types of processes in decision-making (20) based on explicit and implicit attitudes. Explicit and implicit attitudes are our conscious or subconscious assessments of a situation. According to dual-process models, human behavior consists of both controlled (conscious, type-2 process, explicit attitudes) and uncontrolled (unconscious, type-1 process, implicit attitudes) elements (21, 22). If there is an affective-reflective discrepancy and self-control resources are low, behavior is more likely to be determined by the affective type-1 process, the implicit attitudes (19). Siroty et al. (23) recently even reported, that health-favoring implicit food-related associations uniquely contribute to healthier eating behaviors.

### 1.1 The goal of this study

It has been shown that self-control is a prerequisite for sustainable behavior (8) and healthy eating behavior (10). Because explicit attitudes as type-2 processes are highly dependent on self-control (19), the depletion of this resource should exacerbate explicit attitudes toward vegetarian nutrition. For this, the main goal of this study is to show changes in explicit and implicit attitudes toward sustainable behavior (e.g., vegetarian food consumption) through the lack of self-control in a state of ego depletion and to examine their impact on actual sustainable behavior through a decision-making task. This helps to understand the psychological factors of sustainable individual consumption behavior and can increase our understanding of behavioral decisions.

The following hypotheses were formulated:

*H1*: The explicit attitudes toward sustainable vegetarian nutrition will become more negative for the depletion group than the control group (non-depletion group).

*H2*: There are no changes in the implicit attitudes toward sustainable vegetarian or less-sustainable meat-based nutrition in the depletion or non-depletion group.

*H3*: The depletion group will more likely act congruent to their implicit attitudes toward a sustainable vegan or less-sustainable non-vegan chocolate bar than the non-depletion group.

## 2 Methods

### 2.1 Experimental design

A randomized controlled trial (RCT) with the between-subject factor group (ego depletion, non-depletion) and the within-subject factor time (pre- vs. post-intervention) was conducted. Participants completed explicit and implicit evaluation tasks before and after a six-minute transcription task (ego depletion vs. control). Post-intervention, they completed the same evaluation tasks, a demographic questionnaire, and a decision-making task.

### 2.2 Participants

We calculated the required sample size before data collection and expected small effects (24, 25). For an appropriate calculation of the sample size for H1 and H2, we used an open-ended sequential Bayes factor design (26) with a reasonable maximum sample size, calculated in a frequentist way using G*power (27). Using the program JASP, we conducted a Bayesian repeated-measures ANOVA with repeated measures factors (pre/post) and the between-subject factor group with the commonly used and recommended (28) default prior distribution of JASP (*r* scale fixed effects = 0.5; *r* scale random effects = 1; *r* scale covariates = 0.354).

A power analysis, calculated using G*power (27), for a repeated-measures ANOVA with the within-factor time (pre/post) and between-factor group (ego depletion/non-depletion) with a small effect size of *f* = 0.15, an alpha-level of 0.05, a power of 1-ß = 0.95 and a correlation among repeated measurements of 0.5 resulted in *N* = 148 to detect significant differences between the groups in explicit and implicit attitudes toward images of vegetarian and meat-based nutrition. This frequentist sample size rationale indicates a reasonable maximum sample size for a Bayesian repeated-measures ANOVA.

A power analysis using G*power (27) for the one-tailed logistic regression with the probability of choosing the sustainable chocolate bar over the less-sustainable one as the dependent variable and depletion yes/no as the experimental factor with Odds ratio = 2.25 (Pr(y = 1|x = 1)H0 = 0.4), alpha-level of 0.05, a power of 1-ß = 0.8, and a binomial x-distribution resulted in *N* = 155 as the appropriate number of participants for H3.

We expected a dropout rate of approximately 10%. Therefore, the estimated sample size of 155 was increased to 171 subjects, which served as the maximum sample size. As the calculated sample size for H3 is higher than the one for H1 and H2, we used *n* = 171 as the sample size for the whole study. All participants were randomly assigned to one of the two groups (depletion, non-depletion), resulting in equally distributed sample sizes for the groups. Three participants from the final sample of 171 had to be excluded due to more than 50% of non-evaluable trials in the affective priming task.

The study was conducted following the principles of the Helsinki Declaration regarding ethical guidelines and was approved by the Ethical Board of the University of Regensburg (reference number: 20-1978_1–101) and preregistered at OSF: https://osf.io/jgxbn/. The whole sample consisted of students of Applied Movement Science from the University of Regensburg, who were recruited via the institute’s newsletter and received study credits for their participation. The average age was *M* = 22.73, SD = 3.09, with a minimum of 18 and a maximum of 42 years. A total of 140 participants (83.33%) were 10–24 years old, and 28 participants (16.67%) were 25–42 years old. Regarding eating habits, 117 participants (69.64%) are omnivorous, and 51 participants (30.36%) are vegetarian or vegan.

### 2.3 Material

#### 2.3.1 Demographic questionnaire

Participants answered questions concerning gender, age, education stage, the importance of nutrition, the importance of sustainable nutrition, and eating habits (vegan, vegetarian, omnivorous).

#### 2.3.2 Explicit evaluation task

For the explicit rating task, assessing explicit attitudes, five pictures of vegetarian food and five pictures of meat-based food were chosen from the database of Blechert et al. (29), matched in familiarity, arousal, and valence, and one picture of each of the corresponding chocolate bar used in the decision-making task. The explicit evaluation rating task consisted of the following question: “How much do you like the food in the photo?” (1 = “very much,” 7 = “not at all”). Participants had 5 s to respond. Indices are calculated by the mean score of explicit rating for each category. We also recorded the scores for the sustainable and less-sustainable chocolate bars.

#### 2.3.3 Affective priming task

**Figure 1:**
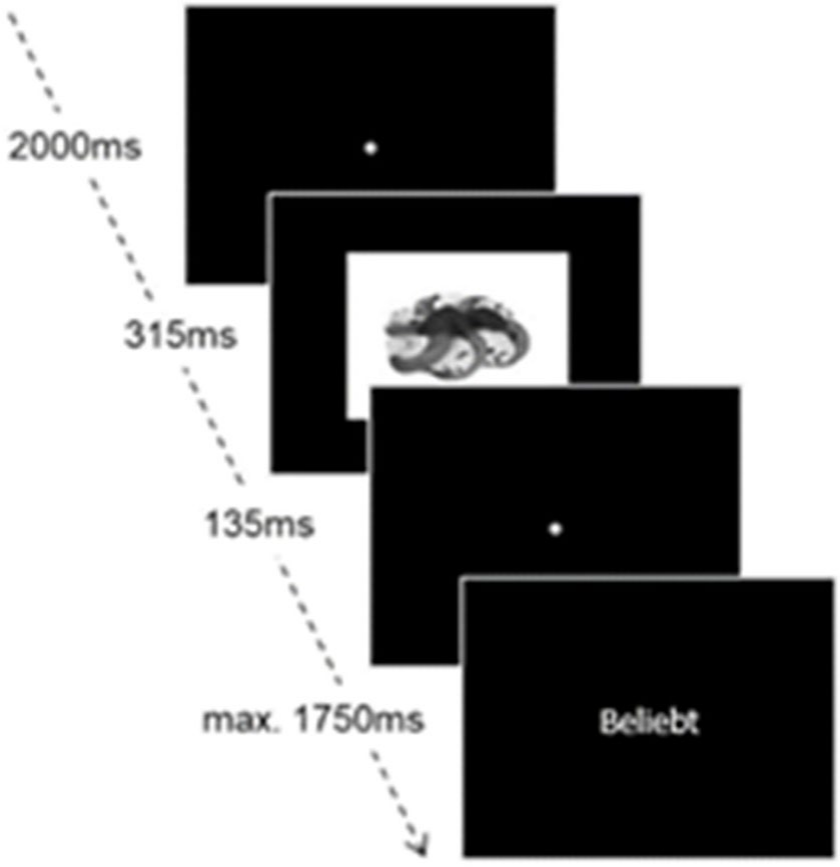
Experimental setting of the affective priming task.

An affective priming paradigm was used to assess the implicit attitudes (30, 31). The same pictures of vegetarian and meat-based food as in the explicit evaluation were used, plus one picture of each chocolate bar used in the decision-making task. Each trial starts with a 2000 ms fixation trial, followed by a picture presentation for a duration of 315 ms. After another 135 ms fixation cross, a positive or negative word [out of a list of four positive and four negative words, retrieved from the Berlin Affective Word List; (32)] is shown, and participants must indicate whether it’s a positive or a negative word using the arrow keys (left = positive; right = negative) in a time window of 1750 ms. Each picture is combined with each word, resulting in 96 trials, one exemplary shown in Figure 1.

An index of implicit attitudes will be calculated from reaction times (31). Reaction times when categorizing picture-primed positive words will be subtracted from reaction times when categorizing picture-primed negative words and will be averaged, respectively, for the categories.

#### 2.3.4 Ego depletion task

Participants perform a transcription task transcribing a neutral text handwritten on a sheet as quickly and accurately as possible for 6 min, omitting the letters “e” and “n” to overcome their usual writing habits, exert self-control, and induce ego depletion. Participants in the non-depletion group do not receive specific instructions on transcribing the text. Following Furley et al. (33), there is a manipulation check afterward to verify that the two transcribing conditions required different levels of self-control strength. This method has been widely used in ego depletion research (34–36), as it effectively exhausts self-control resources by forcing participants to override ingrained response tendencies while providing a consistent cognitive load [unaffected by individual emotional sensitivity with the emotional suppression task; (37)] and is characterized by its task simplicity and feasibility not requiring specialized materials or instructions, making it highly replicable.

#### 2.3.5 Decision-making task

After getting told to have officially finished the study and said goodbye, the instructor offers the participants a chocolate bar, either a “normal” chocolate bar (non-vegan option without any pro-environmental certificate and therefore less-sustainable), or a vegan (sustainable option, proven through a certificate pursuant to article 35(1) of the regulation (EU) 2018/848 on organic production and labeling of organic products by the society for resource protection, documentary number: DE-BE-039-05519-BCD-2022-V1) one, both in same size and type of flavor.

### 2.4 Statistical analysis

Hypotheses and the analytic plan were specified before data collection in the preregistration at OSF: https://osf.io/jgxbn/.

First, demographic variables and important variables for this study are presented.

To test if there are significant differences in explicit and implicit attitudes toward in each case images of sustainable vegetarian and less-sustainable meat-based nutrition, Bayesian repeated-measure ANOVAs were conducted, individually for the dependent variables explicit and implicit attitudes (H1 and H2) and the independent variables group (between, depletion vs. non-depletion) and time (within, pre vs. post).

In the Bayesian repeated-measures ANOVA, the repeated-measures factor is in each case time (pre and post) and the between-subject factor group. The default prior distribution in JASP is used (28). To test H3, a frequentist logistic regression with the probability of choosing the vegan chocolate bar over the non-vegan one as the dependent variable and depletion yes/no as an experimental factor with method Enter was conducted in SPSS. The continuous predictors of implicit and explicit attitudes toward the vegan and non-vegan chocolate bars are added.

Missing data in the affective priming task and explicit evaluation was handled using multiple imputations.

## 3 Results

### 3.1 Demographic data

There is no statistical difference between men and women regarding age [*t*(132.91) = 0.09, *p* = 0.926] and the importance of nutrition [*t*(162.99) = −0.77, *p* = 0.443]. In contrast, the importance of sustainable nutrition is significantly higher for women than men [*t*(161.61) = −2.04, *p* = 0.043]. There is a significant relation between gender and eating habits [*χ*^2^(2) = 15.40, *p* < 0.001], with women’s eating behavior being more often vegetarian or vegan but less omnivore than men’s.

Furthermore, there is no statistical difference between participants of the intervention and the control group regarding age [*t*(166) = −0.47, *p* = 0.319], importance of nutrition [*t*(166) = 0.85, *p* = 0.198], importance of sustainable nutrition [*t*(166) = 0.87, *p* = 0.193], gender [*χ*^2^(1) = 2.82, *p* = 0.093] and eating habits [*χ*^2^(2) = 4.17, *p* = 0.124].

### 3.2 Manipulation check of transcription task

Descriptives of the manipulation check of the transcription task show a significant difference in difficulty, but surprisingly, there are no significant differences in effort and impulse suppression between the two groups, shown in Table 1.

**Table 1:** Descriptives of explicit attitudes toward sustainable vegetarian nutrition (Exp_Veg), implicit attitudes toward sustainable vegetarian nutrition (Imp_Veg), implicit attitudes toward less-sustainable meat-based nutrition (Imp_Meat) and the variables of the manipulation check (effort, impulse suppression, difficulty).

Variable	Time	Group	N	Mean	SD	SE	Coefficient of variation
Exp_Veg	Pre	Ego depletion	86	5.484	0.974	0.105	0.178
		Non-depletion	82	5.588	0.964	0.106	0.172
	Post	Ego depletion	86	5.521	0.973	0.105	0.176
		Non-depletion	82	5.637	0.887	0.098	0.157
Imp_Veg	Pre	Ego depletion	86	22.568	56.393	6.081	2.499
		Non-depletion	82	24.349	52.549	5.803	2.158
	Post	Ego depletion	86	8.299	48.658	5.247	5.863
		Non-depletion	82	9.210	61.387	6.779	6.665
Imp_Meat	Pre	Ego depletion	86	6.230	57.339	6.183	9.203
		Non-depletion	82	17.438	54.879	6.060	3.147
	Post	Ego depletion	86	10.193	48.345	5.213	4.743
		Non-depletion	82	12.971	68.792	7.597	5.303
Effort	Post	Ego depletion	86	4.40	1.43	0.15	2.030
		Non-depletion	82	4.11	1.53	0.17	2.346
Impulse	Post	Ego depletion	86	3.03	2.62	0.28	6.858
		Non-depletion	82	3.04	1.98	0.22	3.937
Difficulty	Post	Ego depletion	86	4.58	1.50	0.16	2.246
		Non-depletion	82	2.27	1.61	0.18	2.594

### 3.3 Explicit attitudes toward sustainable vegetarian nutrition

Using a Bayesian RM ANOVA with the dependent variable explicit attitude toward vegetarian nutrition and the independent variables group (between, ego depletion vs. non-depletion) and time (within, pre vs. post), default prior distribution and half-Cauchy distribution with scale = 0.707 as alternative hypothesis model, the Bayes factor indicates that the data are 0.446 times more likely under the model that includes group, 0.197 times more likely under the model that includes time, 0.089 times more likely under the model that includes time and group and 0.014 times more likely under the model that includes time and group and their interaction as predictors, compared to the null model, as shown in Table 2a. Contrary to H1, the ego depletion group’s explicit attitudes toward sustainable vegetarian nutrition did not become more negative at post-intervention compared to the control group.

**Table 2:** Model comparisons of the Bayesian RM ANOVA with the dependent variables explicit attitude toward sustainable vegetarian nutrition/implicit attitude toward sustainable vegetarian nutrition/implicit attitude toward less-sustainable meat-based nutrition and independent variables group and time.

Models	P(M)	P(M|data)	BF_M_	BF_10_	Error %
(a) Model comparison of the Bayesian RM ANOVA with the dependent variable explicit attitude toward sustainable vegetarian nutrition and independent variables group and time					
Null model	0.200	0.572	5.356	1.000	
Group	0.200	0.256	1.373	0.446	3.660
Time	0.200	0.133	0.509	0.197	1.025
Time + Group	0.200	0.051	0.214	0.089	4.463
Time + Group + Time*Group	0.200	0.008	0.033	0.014	5.727
(b) Model comparison of the Bayesian RM ANOVA with the dependent variable implicit attitude toward sustainable vegetarian nutrition and independent variables group and time					
Null model	0.200	0.080	0.346	1.000	
Time	0.200	0.757	12.439	9.513	1.114
Time + Group	0.200	0.127	0.584	1.602	1.280
Time + Group + Time*Group	0.200	0.022	0.090	0.276	2.528
Group	0.200	0.014	0.059	0.181	4.586
(c) Model comparison of the Bayesian RM ANOVA with the dependent variable implicit attitude toward less-sustainable meat-based nutrition and independent variables group and time					
Null model	0.200	0.703	9.484	1.000	
Group	0.200	0.186	0.913	0.264	0.744
Time	0.200	0.084	0.365	0.119	1.350
Time + Group	0.200	0.022	0.090	0.031	1.369
Time + Group + Time*Group	0.200	0.005	0.021	0.007	2.692

### 3.4 Implicit attitudes toward sustainable vegetarian and less-sustainable meat-based nutrition

Using a Bayesian RM ANOVA with the dependent variable implicit attitude toward sustainable vegetarian nutrition and the independent variables group (between, depletion vs. non-depletion) and time (within, pre vs. post), default prior distribution and half-Cauchy distribution with scale = 0.0707 as alternative hypothesis model, Table 2b shows that the Bayes factor indicates the data are 9.513 times more likely under the model that includes time, 1.602 times more likely under the model that includes time and group, 0.276 times more likely under the model that includes time and group and their interaction and 0.181 times more likely under the model that includes group as predictor, compared to the null model. Contrary to H2, the implicit attitudes toward sustainable vegetarian nutrition became more negative, comparing pre- and post-intervention. However, in line with our theory, this more negative implicit valuation did not differ between the groups.

Using a Bayesian RM ANOVA with the dependent variable implicit attitude toward less-sustainable meat-based nutrition and the independent variables group (between, depletion vs. non-depletion) and time (within, pre vs. post), default prior distribution and half-Cauchy distribution with scale = 0.0707 as alternative hypothesis model, the Bayes factor shown in Table 2c indicates that the data are 0.264 times more likely under the model that includes group, 0.119 times more likely under the model that includes time, 0.031 times more likely under the model that includes time and group and 0.007 times more likely under the model that includes time and group and their interaction as predictors, compared to the null model. As implicit attitudes toward less-sustainable meat-based nutrition did also show no group difference, like the implicit attitudes toward sustainable vegetarian nutrition, the part of the H2 can confirm that the amount of remaining self-control has no impact on type-1 processes and implicit attitudes.

**Figure 2:**
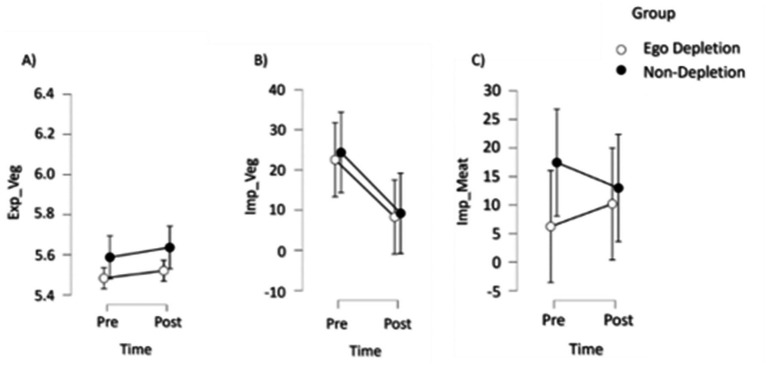
Descriptives plots (Mean, SE) of **(A)** explicit attitudes toward sustainable vegetarian nutrition (Exp_Veg), **(B)** implicit attitudes toward sustainable vegetarian nutrition (Imp_Veg) and **(C)** implicit attitudes toward less-sustainable meat-based nutrition (Imp_Meat).

All descriptives of implicit and explicit attitudes measurements and the manipulation check can be found in Table 1, descriptive plots of attitude measurements are shown in Figure 2.

### 3.5 Effect of attitudes on actual behavior

The binomial logistic regression model with the probability of choosing the sustainable chocolate bar over the less-sustainable one as dependent variable and depletion yes/no as experimental factor with the method Enter and the continuous predictors implicit and explicit attitudes were statistically significant, *χ*^2^(13) = 49.886, *p* < 0.001, resulting in an acceptable amount of explained variance (38), as shown by Nagelkerkes’ *R*^2^ = 0.396. Goodness-of-fit was assessed using the Hosmer-Lemeshow-Test, indicating a good model fit, *χ*^2^(8) = 7.08, *p* > 0.05. Overall percentage of accuracy in classification was 80.3%, with a sensitivity of 47.6% and a specificity of 92.2%. Of the 13 variables entered into the regression model, only one contributed significantly to predicting the choice of a sustainable chocolate bar: explicit attitude toward the sustainable chocolate bar after the intervention (Exp_VeganzPost, *p* = 0.041), while the other variables showed no significant effect. Higher Exp_VeganzPost implies an increased likelihood of choosing the sustainable chocolate bar, OR = 0.591 (95% CI [1.025, 3.177]). All model coefficients and odds can be found in Supplementary Table 1. These do not confirm H3 as the only variable implying an increased likelihood of choosing the sustainable chocolate bar is the explicit attitude toward the sustainable chocolate bar at post-intervention measurement.

## 4 Discussion

This study aimed to show changes in explicit and implicit attitudes toward sustainable behavior caused by a lack of self-control in a state of ego depletion and examine their impact on actual sustainable behavior.

### 4.1 Self-control and explicit attitudes toward sustainable vegetarian nutrition

The ego depletion group’s explicit attitudes toward sustainable vegetarian nutrition did not become more negative post-intervention than the control group. In fact, both groups’ explicit attitudes toward sustainable vegetarian nutrition did become more positive from pre- to post-intervention to a nearly similar extent.

Because of this, the assumption could not be confirmed that the lack of self-control in the ego depletion group at post-intervention leads to disregard of positive aspects of sustainability aspects, like values, norms, and morals, or sticking to long-term goals, and therefore, without these mentioned aspects to a more negative explicit rating of sustainable vegetarian nutrition. Not only was there no detectable difference between the two groups, indicating that the amount of remaining self-control has no impact on the rating of explicit attitudes, but they even became more positive in the intervention group than in the control group. Therefore, the state of ego depletion, resulting in a short-term limitation of one’s own beliefs in values, norms, morals, and long-term goals, does not affect the explicit evaluation of sustainable vegetarian food, as expected by the theory of ART (19) and contradictory to H1. The reasons for this are speculative.

One speculative assumption is that the explicit evaluations seem too stable and firmly anchored in the everyday life of this sample to get affected by a single, 6-minute intervention. This sample, composed only of sports students, is primarily aware of healthy nutrition. A non-negligible proportion of them have healthy and vegetarian eating habits and therefore have a biased, familiar, and firmly anchored positive view on vegetarian food, as especially in western societies, eating meat has lately been more and more criticized due to environmental, health-related, or humanitarian concerns (39). This is consistent with findings from Ghaffar & Islam (40), who identified health consciousness and social pressure as stable drivers of sustainable behavior among millennials, suggesting that core values and social identity may buffer against short-term ego depletion effects.

### 4.2 Self-control and implicit attitudes toward sustainable vegetarian and less-sustainable meat-based nutrition

The implicit attitudes toward sustainable vegetarian nutrition did become more negative, comparing pre- and post-intervention. This more negative implicit valuation did not differ between the groups, which aligns with the theory that self-control and, therefore, the effect of ego depletion does not affect type-1 processes and its automatic affective valuation, implicit attitudes. As the implicit attitudes toward less-sustainable meat-based nutrition did also show no group difference, like the implicit attitudes toward sustainable vegetarian food, part of the H2 can be confirmed that the amount of remaining self-control has no impact on type-1 processes based on implicit attitudes, which is in line with the theory of the ART (19).

A possible explanation for the divergence between explicit attitudes toward vegetarian food, which became more positive, and implicit attitudes, which became more negative, is cognitive dissonance (41). Participants might have been aware of the study’s sustainability focus and consciously adjusted their explicit attitudes in a socially desirable direction. In contrast, implicit attitudes, driven by automatic associations, remained unaffected by rationalization and even became more negative.

### 4.3 Self-control and sustainable decision-making

The ego depletion group with no remaining self-control was expected to choose the chocolate bar congruent to their implicit attitudes toward it, as according to Brand and Ekkekakis (19), the type-2 process without self-control is impossible.

The results of this study do not confirm this hypothesis as the only variable implying an increased likelihood of choosing the sustainable chocolate bar is the explicit attitude toward the sustainable chocolate bar at post-intervention measurement, which is an interesting finding of this study. The actual real-life sustainable behavior, according to the ART, is either determined by the type-2 process based on explicit attitudes or the type-1 process based on implicit attitudes. This study defines the type-2 process with its explicit aspects of values, norms, morals, and long-term thinking as the predominant influences on sustainable behavior, contrary to Siroty et al. (23), who attributed implicit attitudes as the sole contributor to eating behavioral change.

The state of ego depletion did not have the power to shut off all of the type-2 processes, which still did play the most prominent role in this decision-making task. The reasons for this are speculative. The effect of ego depletion could have been weakened through social goals and cash incentives (7). The participants were asked to choose the chocolate bar after they were told the experiment was over. Nonetheless, it cannot be excluded that some of the participants took the sustainable chocolate bar because they assumed the topic of the study was related to sustainability and wanted to help the investigator get good results. Cash incentives could have been triggered because the sustainable chocolate bar costs 1.95€ compared to the less-sustainable one at 0.48€, which some participants might have been aware of.

### 4.4 Limitations and future research

As far as we know, this is the first study to investigate the implicit and explicit attitudes toward sustainable nutrition in the context of ART (19) and the effect of ego depletion, especially with the additional real-world decision-making task under ego depletion. The RCT design offers several key advantages that strengthen the validity and reliability of the results.

A limitation is the missing differentiation of meat-free food and complete plant-based food, and there are no possible investigations on existing differences in the motivational and psychological profiles of vegetarians and vegans (42).

Another limitation of this study is the length of the affective priming task, which is around 6 min. This length of a concentration period is exhausting and might have also depleted the self-control resources of the control group without the ego-depleting transcription task.

The lack of difference between these two groups can also be explained by the handwritten transcription task, as the validity of the ego depletion manipulation remains a limitation. While the manipulation check confirmed differences in task difficulty, there were no significant differences in effort and impulse suppression between groups, as students aren’t used to handwriting nowadays. This raises concerns about whether the depletion task effectively reduced self-control.

Moreover, the time between the ego depletion task and the decision task was filled with the explicit evaluation, the affective priming task, and the demographic questionnaire, which could be a too long period of time. The self-control resource could be recovered again at the decision-making task.

Furthermore, the sample predominantly consists of younger sports students, who are probably more aware of healthy and vegetarian nutrition and have a more positive and firmly anchored explicit evaluation of sustainable vegetarian nutrition than the average population. Additionally, self-control and vulnerability to ego depletion can vary with age, as older adults tend to have greater self-regulatory capacities and may be less affected by depletion effects (7, 43).

Therefore, future studies should investigate different items in the decision-making task with no cash incentives, evaluate a more variational sample, use a shorter method of evaluating implicit attitudes, and use a computer-based transcription task.

## 5 Conclusion

This study does neither show an effect of ego depletion on explicit nor implicit attitudes toward sustainable or less-sustainable nutrition, which suggests that self-control is no main psychological factor in sustainable behavior and adds to the literature that questions the effect of ego depletion (44). However, the results should be dealt with carefully, as this study is also an indication that the transcription task should not be executed in a handwritten way.

Regarding the ART, this study indicates that self-control and the state of ego depletion have no impact on implicit attitudes and identifies type-2 processes, explicit attitudes, as the predominant influence on explaining and describing sustainable behavior compared to type-1 processes, implicit attitudes. This emphasizes the importance of spreading and strengthening the foundations of explicit evaluations of sustainable behavior, like the availability of relevant propositional information, forming pro-environmental ideals, values, morals, social expectations, or the will to reach long-term goals to empower sustainable behavior.

## Data Availability

The datasets presented in this study can be found in online repositories. The names of the repository/repositories and accession number(s) can be found at: OSF storage https://osf.io/t9nqy.
