# Supplementary material for: Ego depletion and its role regarding the attitudes and behavior toward sustainable food consumption
Source: Front Nutr. 2025 May 8;12:1469301. doi: 10.3389/fnut.2025.1469301 (PMC12095011; doi:10.3389/fnut.2025.1469301)
Supplement: Supplementary file 1 [file Table_1.docx]

Supplementary Table 1

Variables of the binomial logistic regression model with the probability to choose the sustainable vegan chocolate bar over the less-sustainable non-vegan one as dependent variable and group as experimental factor

|  | *B* | | *SE* | | *P* | | Odds Ratio | | 95% CI for Odds Ratio | | | | |  |
| --- | --- | --- | --- | --- | --- | --- | --- | --- | --- | --- | --- | --- | --- | --- |
|  | | | | | | | | | Lower Bound | | Upper Bound | | |  |
| IntGrImpSniPre | -0.005 |  | 0.005 | .269 | | 0.995 | | 0.986 | | 1.004 | | |  | |
| IntGrImpSniPost | -0.005 |  | 0.006 | .385 | | 0.995 | | 0.983 | | 1.007 | | |  | |
| IntGrImpVeganzPre | 0.001 |  | 0.005 | .809 | | 1.001 | | 0.992 | | 1.010 | | |  | |
| IntGrImpVeganzPost | 0.002 |  | 0.006 | .740 | | 1.002 | | 0.990 | | 1.014 | | |  | |
| Implicit_SnickersPre | 0.002 |  | 0.003 | .533 | | 1.002 | | 0.996 | | 1.007 | | |  | |
| Implicit_SnickersPost | 0.007 |  | 0.004 | .086 | | 1.007 | | 0.999 | | 1.015 | | |  | |
| Implicit_VeganzPre | -0.001 |  | 0.003 | .637 | | 0.999 | | 0.992 | | 1.005 | | |  | |
| Implicit_VeganzPost | 0.000 |  | 0.003 | .886 | | 1.000 | | 0.994 | | 1.007 | | |  | |
| Exp_SnickersPre | -0.398 |  | 0.224 | .076 | | 0.672 | | 0.433 | | 1.042 | | |  | |
| Exp_SnickersPost | -0.319 |  | 0.221 | .149 | | 0.727 | | 0.471 | | 1.121 | | |  | |
| Exp_VeganzPre | 0.370 |  | 0.272 | .173 | | 1.488 | | 0.850 | | 2.467 | | |  | |
| Exp_VeganzPost | 0.591* |  | 0.288 | .041 | | 1.805 | | 1.025 | | 3.177 | | |  | |
| Group | 0.469 |  | 0.487 | .335 | | 1.599 | | 0.616 | | 4.150 | | |  | |
| Constant | -0.090 |  | 0.725 | .902 | | 0.914 | |  | |  | |  | | |
